# Detection of Typical Compensatory Movements during Autonomously Performed Exercises Preventing Low Back Pain (LBP)

**DOI:** 10.3390/s22010111

**Published:** 2021-12-24

**Authors:** Asaad Sellmann, Désirée Wagner, Lucas Holtz, Jörg Eschweiler, Christian Diers, Sybele Williams, Catherine Disselhorst-Klug

**Affiliations:** 1 Department of Rehabilitation and Prevention Engineering, Institute of Applied Medical Engineering, RWTH Aachen University, 52074 Aachen, Germany; desiree.wagner@rwth-aachen.de (D.W.); lucas.holtz@rwth-aachen.de (L.H.); williams@ame.rwth-aachen.de (S.W.); disselhorst-klug@ame.rwth-aachen.de (C.D.-K.); 2 Department of Orthopaedics, Trauma and Reconstructive Surgery, RWTH Aachen University Clinic, 52074 Aachen, Germany; joeschweiler@ukaachen.de; 3 Diers International GmbH, 65388 Schlangenbad, Germany; chris@diers.de

**Keywords:** low back pain, rehabilitation, motion analysis, wearable sensors, accelerometer, biomechanics, feature extraction, pattern recognition

## Abstract

With the growing number of people seeking medical advice due to low back pain (LBP), individualised physiotherapeutic rehabilitation is becoming increasingly relevant. Thirty volunteers were asked to perform three typical LBP rehabilitation exercises (Prone-Rocking, Bird-Dog and Rowing) in two categories: clinically prescribed exercise (CPE) and typical compensatory movement (TCM). Three inertial sensors were used to detect the movement of the back during exercise performance and thus generate a dataset that is used to develop an algorithm that detects typical compensatory movements in autonomously performed LBP exercises. The best feature combinations out of 50 derived features displaying the highest capacity to differentiate between CPE and TCM in each exercise were determined. For classifying exercise movements as CPE or TCM, a binary decision tree was trained with the best performing features. The results showed that the trained classifier is able to distinguish CPE from TCM in Bird-Dog, Prone-Rocking and Rowing with up to 97.7% (Head Sensor, one feature), 98.9% (Upper back Sensor, one feature) and 80.5% (Upper back Sensor, two features) using only one sensor. Thus, as a proof-of-concept, the introduced classification models can be used to detect typical compensatory movements in autonomously performed LBP exercises.

## 1. Introduction

Rehabilitation is usually performed in a hospital or outpatient environment. Exercising for preventing physical disorders in contrast is mostly autonomously performed at home or at a gym. Physiotherapy is the main path to follow when focusing on orthopaedic disorders and especially low back pain (LBP). The global Years Lived with Disabilities (YLDs) caused by LBP between 1990 and 2017 increased by 52.7% to 64.9 million. In the case of LBP, Western Europe had the highest number of YLDs and LBP is a common reason for a medical consultation [1,2,3]. It has been shown that exercise not only reduces pain and increases function in patients with low back pain [4] but also generally increases individuals’ well-being [5]. Thus, it is highly desirable for patients with LBP to increase the hours spent performing rehabilitation exercises. The exercises typically prescribed in rehabilitation are designed to optimise muscle activation and coordination. Therefore, it is important that patients closely adhere to instructions, especially when performing exercises autonomously to reduce pain and restore the quality of a patients’ life, as much as possible. However, 70% of patients do not conscientiously follow prescribed exercise plans [6], which reduces the effectiveness of rehabilitation measures.

In the absence of accompanying clinical guidance and control during autonomous exercise, patients may face a higher risk of injury through compensatory movements, e.g., lumbar rotation [7]. Hence, methods to monitor the execution of rehabilitation exercises outside of clinical or rehabilitation environments are needed. These will minimise secondary injury related to incorrect exercise performance.

Van Dijk et al. outlined that healthcare professionals recognise the importance of a standardised way of observing movement quality, but a tool or assessment that could offer this kind of functionality, does not exist yet [8]. With the miniaturization of wearable technologies such as the development of microelectromechanical systems (MEMS), it has become feasible to use such systems in clinical assessments and especially in home- or gym- environments. Several researchers have examined the feasibility of using systems based on inertial sensors to objectively study human movement and thus provide objective tools to measure and assess exercise activity [9,10,11,12,13,14]. Most of the researchers concluded that inertial sensor systems are sufficient for the detection of human movement and superior when it comes to portability and usability, especially when compared to optoelectronic marker-based or markerless systems [15,16,17,18].

Other research groups have contributed to the field of patient monitoring by applying machine learning to monitoring several rehabilitation exercises and focusing on different parts of the body [9,10,11,12,19,20,21,22]. Bavan et al. evaluated the feasibility of using a single inertial sensor to recognise and classify shoulder rehabilitation activity using a support vector machine (ten-fold cross validation: 97.2%) and random forests (leave-one-subject-out-validation: 80.5%) [9]. Mannini et al. focused on the classification of human motion in general using Hidden Markov Models [12]. While Dan Morris et al. focused on strength training and repetitive exercises in general, using support vector machines to employ segmenting, recognizing and counting with precision and recall greater than 95% in identifying exercise periods, recognition of 99%, 98% and 96% on circuits of 4, 7 and 13 exercises respectively [10].

Ranganathan et al. identified compensatory trunk movements during reaching tasks in the upper extremity using three wearable sensors positioned on trunk (chest), upper arm and forearm. To validate their results an 8-camera motion capture system was used to determine ground truth. Using 10-fold cross validation their algorithms reached a precision and recall of 88.6% and 91.2% respectively, using two features. These features were the standard deviation of 1: the acceleration along the Z-axis (perpendicular to the chest) in the trunk sensor and 2: the angular velocity along the Z-axis (dorsal direction) in the forearm sensor. They were thus able to show that compensatory movements in the trunk can be detected with acceleration sensors during reaching tasks. [23] Eizentals et al. analysed 11 exercises with specific compensatory movements using textile stretch sensors on a shirt. They were able to demonstrate the ability of the shirt to detect minor movement differences. [24] Barth et al. conducted an analysis to characterize how accelerometer variables reflect upper limb compensatory movement patterns after stroke. They confirmed that accelerometry is a tool that can reflect the use of general compensatory movement patterns of the upper limb in persons with chronic stroke. Moreover, they found that out-of-clinic measurements had stronger relationships with compensatory movements compared with in-clinic measurements. [25]

LBP specific rehabilitation and prevention exercises are predestined to be monitored using inertial sensors due to their low performance speed and repetitive nature. Such systems have already been employed by Peng et al. to quantitatively analyse spine angle range during dynamic exercises to provide an objective reference of disability level of LBP patients [26]. Matijevich et al. presented a wearable approach for monitoring low back loading during manual material handling using pressure insoles and inertial measurement units (IMUs) [27]. Furthermore, motion detection systems have been used to support the rehabilitation of a variety of musculoskeletal diseases such as knee disorders and low back pain [28,29]. For example, de Villa et al. were able to assess the performance of rehabilitation exercises implementing a lower limb joint angle measurement system [30]. The most frequently used technology for these motion detection systems are accelerometers [31,32].

Combined with state-of-the-art machine learning procedures, these low-cost systems are the perfect choice for a system to guide and control LBP patients in their performance of rehabilitation or prevention exercises. Therefore, the motivation for this work is to develop a method to detect compensatory movements in rehabilitation and prevention exercises in community settings that is easy to use for both patients and practitioners and thus prevent patients from movements that lead to a deterioration of their condition.

## 2. Materials and Methods

The method to be introduced aims to automatically detect the most typical, critical compensatory movements in autonomously performed rehabilitation and prevention exercises. According to Table 1➀, experts were conducted to define the clinically prescribed way of performing exercises and the corresponding typical compensatory movements. In ➁ a dataset was generated, which was then processed to generate and validate a classification model. For validation, it was important to use the leave-one-subject-out method to present absolutely unknown data to the classification model. The summarised procedure in Table 1 is further explained in the following paragraphs.

### 2.1. Preparation

In collaboration with physiotherapists and orthopaedic surgeons from the RWTH Aachen University Clinic, Germany, a catalogue of 27 exercises typically prescribed to patients for continuous rehabilitation after suffering acute low back pain was analysed. The catalogue of exercises originates in a database that is used to foster a rehabilitation plan for patients based on their diagnosis. The analysis included the discussion of the feasibility and efficacy of these exercises for patients with different degrees of low back pain. Focusing on quality of exercise performance to reach the best rehabilitation results, the experts discussed the most typical compensatory movements, especially in autonomous training where patients perform these exercises in community settings.

For the presented study a set of three exercises out of this catalogue was chosen as a representation for clinically prescribed exercises to patients after suffering acute low back pain. These three exercises were: Prone-Rocking, Bird-Dog and Rowing.

### 2.2. Generating Datasets

#### 2.2.1. Participants

Thirty healthy subjects (15 male/15 female) without a history of back pain participated in this study. The age of the subjects ranged from 18 to 35 years with an average age of 27.4 ± 2.5 years. All participants regularly participated in exercise, this resulted in a good body awareness and thus increased the likelihood that subjects were able to perform TCMs and CPEs as instructed. The risk of injury while performing the exercises in the study was low at all times. The following inclusion and exclusion criteria (Table 2) were used as the basis for the selection of subjects.

The study was approved by the Human Ethics Committee of RWTH Aachen University, and all subjects were fully informed about all possible occurrences prior to the study (EK 134-19) and gave informed consent.

#### 2.2.2. Data Acquisition

**Figure 1:**
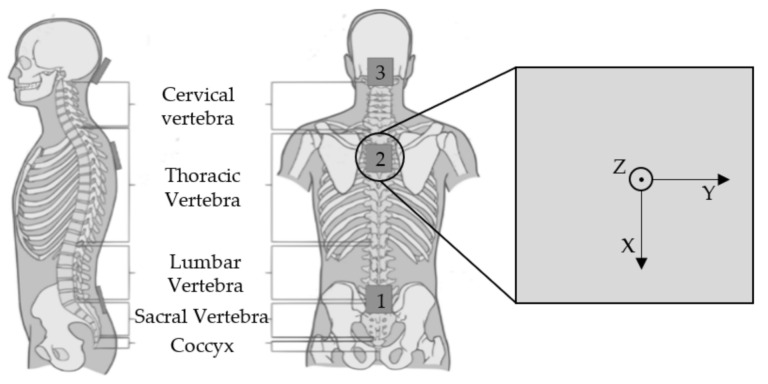
Positioning of the sensors: (**1**) between the fifth lumbar-vertebra (L5) and the first sacral-vertebra (S1), (**2**) at the transition from thoracic- to cervical-vertebra (Th1, C7) and (**3**) on the back of the head. On the right, the axes of the sensors are depicted.

Three accelerometers (Bosch BMX055, 200 Hz sampling frequency, 12-bit resolution, gyroscope and magnetometer turned off) were used to record accelerations associated with movement of the back during exercise execution. According to Figure 1, the sensors were placed at three sites along the spine; (1) between the fifth lumbar vertebra (L5) and the first sacral vertebra (S1), (2) at the transition from thoracic to cervical-vertebra (Th1, C7) and (3) on the back of the subjects’ head. Sensor 3 was mounted on an adjustable headband so it could be securely positioned. The X-axis of the sensors was roughly aligned with the longitudinal axis of the subject’s body heading from cranial to caudal. The Y-axis of the sensors is aligned in a way that it is parallel to the transversal axis of the body (lateral to medial). Finally, the Z-axis of the sensors is roughly aligned parallel to the sagittal axis of the body which heads from ventral to dorsal.

The volunteers were asked to perform the selected exercises in two categories: clinically prescribed exercise (CPE) and a typical compensatory movement (TCM). CPEs were optimised to ensure proper form and the TCMs were designed to reflect typical compensatory movements. TCMs were according to the analysis in corporation with experienced physiotherapists from the University Clinic in Aachen, Germany. Prior to the measurements the order of the categories CPE, TCM and the exercises within each category were randomised for each subject.

**Figure 2:**
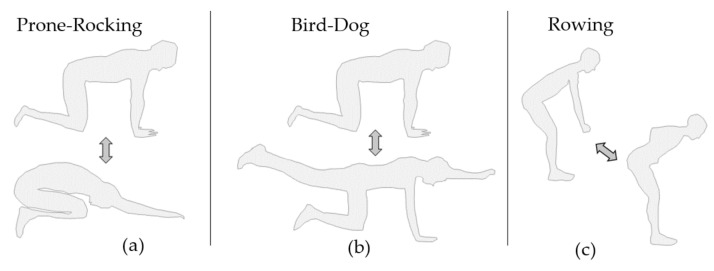
CPE: Exercises performed in clinically prescribed optimised form. (**a**) Prone-Rocking, (**b**) Bird-Dog and (**c**) Bent-over Rowing.

As indicated in Figure 2a, when performing Prone-Rocking, the subject started from a quadruped stand and then moved the upper body backwards until the gluteus touched the heels of the feet, following Voight et al. [33]. Hands, knees and feet remained in position on the ground during exercise performance. Bird-Dog, Figure 2b also started in quadruped stand, but out of the start position the subject then lifted one arm and the diagonally opposed leg. Hand, arm and leg formed a straight line with spine and head following Graham et al. [34]. Figure 2c depicts the exercise Bent-over Rowing. The subject started slightly bent forward with the arms hanging downwards, as if holding an imaginary weight. The imaginary weight was pulled towards the navel until both hands touched the sides of the body. Hand and arm segments of both sides stayed parallel during execution. Standing Bent-over rowing follows the instructions by Fenwick et al. in [35].

For each of the two categories three sets of six repetitions of each exercise were performed. The test subjects were carefully instructed by the test supervisor. As Bird-Dog was performed on both sides, each subject performed a total of 24 sets (2 categories × 4 exercises × 3 sets).

Due to the design of this study both categories CPE and TCM had the same number of data points after pre-processing.

#### 2.2.3. Measurement Procedure

Three sensors were attached to each individual, using clinically-tested, double-sided, adhesive tape (Typ T06, Nr. 65.2006.00, tyco Healthcare, 2007). Once the order of the categories and exercises was randomly chosen, subjects were asked to get into the exercise’s specific starting position. During Bird-Dog and Prone-Rocking the test subjects used an exercise mat to reduce loading on knees and hands. To identify the start and end of each set and after each repetition within a set, the test supervisor generated an analogue signal by activating a push-button.

### 2.3. Data Processing

#### 2.3.1. Signal Pre-Processing

As described above, each subject performed three sets of an exercise per category (CPE/TCM) with six repetitions per set. Sets were subdivided into repetitions using the analogue signals introduced by the investigator (via a push-button).

The data was normalised to the duration of a fully executed repetition for the respective set in order to make fast and slow movements within a set comparable with each other. Hence, the longest of all six repetitions in terms of time is taken as the reference with 100% duration. Acceleration data was then averaged over the repetitions of one set to account for outliers. This procedure resulted in 180 data points per exercise (30 test subjects × 2 categories × 3 sets).

#### 2.3.2. Feature Extraction

**Figure 3:**
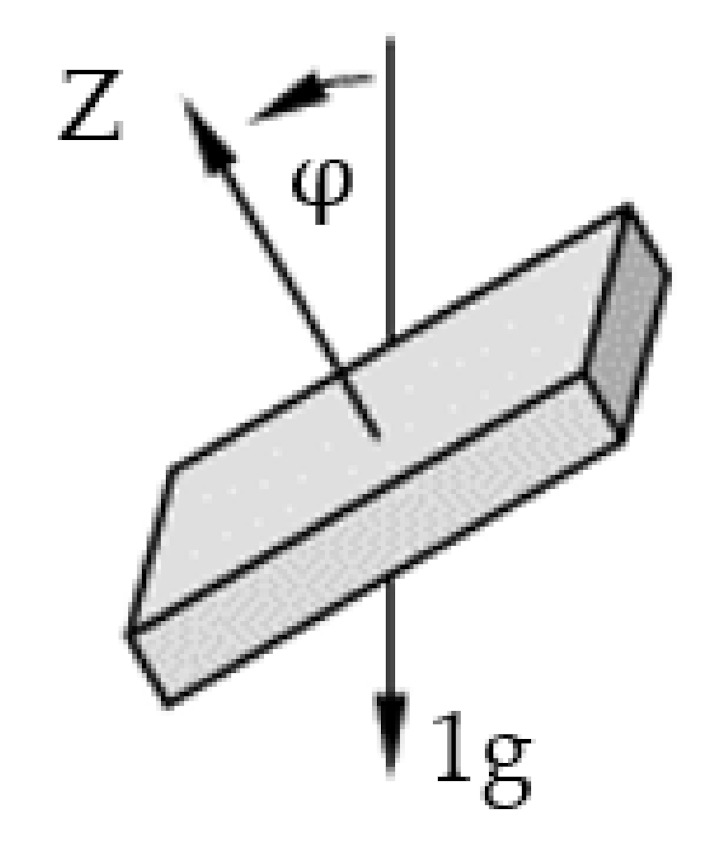
Angle of the sensor with respect to gravity in spherical coordinate system.

Pattern recognition is most commonly performed on a representation of the data sets using parameters as features instead of the raw data sets. Therefore, the pre-processed accelerometer signals were used to calculate a series of features, based on each of the nine signals arising from the X-, Y- and Z-axis of the three sensors. The features deduced from the signals are the maximal angles between Sensor 1 and 2 as well as Sensor 2 and 3. First, the orientation of the sensors i.e., the tilt angle, φ between the Z-axis of the sensor and its gravity vector (Figure 3) was determined using Equation (1) [36]:

(1) $$\varphi =\cos^{-1}\left(\frac{A_{Z}}{\sqrt[{2}]{A_{X}^{2}+A_{Y}^{2}+A_{Z}^{2}}}\right)$$

where A_X_, A_Y_ and A_Z_ represent the linear accelerations in the X-, Y- and Z-directions. Having determined the tilt-angles for all of the three sensors, the difference between tilt angles of Sensors 1 and 2 as well as Sensors 2 and 3 was calculated using subtraction. Determining the maximum in angle deltas results in max(∆φ_1,2_) and max(∆φ_2,3_).

Furthermore, the statistical parameters that were used to evaluate the time-normalised signals were root mean square of accelerations (aRMS) and tilt angles (φRMS), maxima (max), skewness (s), kurtosis (k) and variance (σ^2^). As a standard and well established feature the root mean square (RMS) is defined as follows:

(2) $$\mathrm{aRMS}=\sqrt[{2}]{\frac{1}{N_{a}}\sum_{1}^{N_{a}}a_{i}^{2}},\;\varphi \mathrm{RMS}=\sqrt[{2}]{\frac{1}{N_{\varphi }}\sum_{1}^{N_{\varphi }}\varphi_{i}^{2}}$$

where N is the number of instances (acceleration or tilt angles) and a or φ the value of acceleration or tilt angle at index, i. The variance, $\sigma^{2}$ is defined in Equation (3), where  n ¯ is the mean value of all accelerations.

(3) $$\sigma^{2}=\frac{\sum_{1}^{N}{\left(n_{i}-\;n\;¯\right)}^{2}}{N}$$

σ denotes the standard deviation for the following equations. Skewness and kurtosis are defined in Equations (4) and (5) respectively, where E (t) represents the expected value of the quantity t.

(4) $$s=\frac{E{\left(n-\bar{n}\right)}^{3}}{\sigma^{3}}$$

(5) $$k=\frac{E{\left(n-\bar{n}\right)}^{4}}{\sigma^{4}}$$

All features calculated on each set were then tabulated for each exercise containing subject-, CPE-/TCM- and set-identifiers. Each feature is normalised using the z-score (Equation (6)).

(6) $$z=\frac{\left(n-\bar{n}\right)}{\sigma }$$

The completed table is called the Feature Matrix and contains 50 features (5 Parameters from 9 signals and max(∆φ_1,2_) and max(∆φ_2,3_), ( φRMS1, φRMS2 and φRMS3).

#### 2.3.3. Feature Selection

It is important to consider all combinations of features when checking features in terms of their predictive power. Therefore, random subsets of feature combinations were tested instead of every single feature by itself [37]. The maximum number of features per subset has to be smaller or equal to $\sqrt[{2}]{N}$, where N is the number of data points used for the training of the classifier. This is general practice to avoid overfitting a classification model.

**Figure 4:**
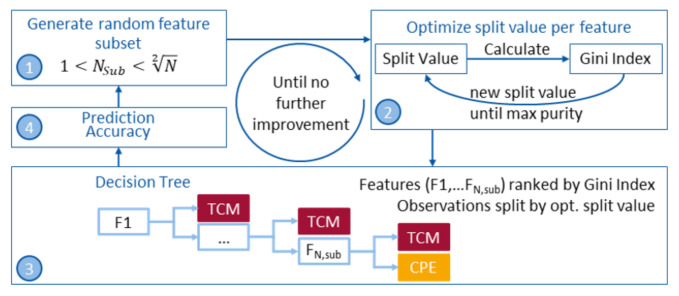
Forward Feature Selection Process. Sequentially adding features to the feature subset until no further improvement in prediction accuracy is gained.

In general, there are three different approaches to selecting features; filter-, embedded- and wrapper-methods. For the presented work, a wrapper method (forward feature selection) was used to find the best performing subset of features. In wrapper methods, the feature selection is wrapped around the classification model and the prediction accuracy of the model is used to iteratively select or eliminate a subset of features [38]. This approach starts with an empty subset of features and sequentially adds features to the subset until there is no further improvement in prediction (see Figure 4). For each feature in a subset, the split value is optimised using Gini’s index [39] to gain the maximum in purity for a node. The corresponding decision tree is then built and the prediction accuracy is obtained using a 5-fold-cross-validation. N, the number of observations used for each training process, is 36 and the maximum number of features per subset N_Sub_ < 6.

With the maximum number of 5 features there are 2,369,935 possible combinations:

$$\sum_{k=1}^{5}\left(\begin{array}{c} N\\ k \end{array}\right)=2369935,\;N=50$$

A loss function to calculate the misclassifications of each decision tree is used as the stop criterion. When there is no further improvement in loss generated by a tree, the algorithm stops sequentially adding further features. That way, having to use all combinations for building decision trees is avoided, which would be computationally very inefficient and time consuming. In the end a maximum of 6, 8 and 15 different subset combinations for Bird-Dog, Prone-Rocking and Rowing respectively had to be calculated, until the minimum in loss was reached.

The prediction accuracy is the sum of correct classified observations, i.e., true CPE and true TCM, divided by the total number of observations. Following the prediction accuracy, the best performing subset of features is used to train the final classification model for each exercise.

To analyse if it was possible to reduce the number of sensors while maintaining a suitable prediction accuracy, all sensors were tested as single-sensor-systems. That reduced the total number of features to 16 (5 parameters on 3 axes per sensor plus φRMS per sensor) and thus a maximum number of 6884 possible combinations for feature subsets to be tested. For this task, a maximum of 9, 10 and 20 different subset combinations for Bird-Dog, Prone-Rocking and Rowing respectively had to be calculated, until the minimum in loss was reached.

#### 2.3.4. Classification Models

An algorithm for classification in supervised learning basically uses known (labelled) data to learn about reality and from there predict unknown (unlabelled) data. Predictions can only be made about classes that were present in the training dataset. So, the trained algorithm that maps the new input to a specific class learned from the training data is called classifier.

As the presented work aims to solve a binary decision problem, decision trees are the first choice. Additionally, decision trees were used mainly because they are considered easily comprehensible due to their graphical structure and because they contain a subset of features rather than using overwhelmingly large numbers [40,41]. This is particularly important because classification results in medical applications have to be explainable to mostly medical staff.

To predict a response, one can follow the decisions in the tree from the root, the first node, down to a leaf node. For these reasons, the use of decision trees to solve the binary decision problem of distinguishing between CPE and TCM was chosen.

As mentioned in Section 2.3.3, the best performing set of features was used to train a decision tree based classifier for each exercise. To create the decision tree for classification, the standard CART algorithm [42] by Breiman et al. was used.

The root node, also called parent node, contains the whole training data. These are then split at a determined value for the first feature. Thus, producing two child nodes with each higher purity than the parent node in terms of contained classes. The purity of a leaf node is representative for how mixed the training data assigned to that node is. To optimise the purity the Gini index [39] was used. According to Equation (7), where G is the Gini index over all classes and p_k_ is the proportion of training instances of a certain class k within the node of interest, G = 0 would be a perfect class purity and G = 0.5 would be an equal distribution of classes (binary problem).

(7) $$G=1-\underset{k}{\sum }p_{k}{}^{2}$$

Basically, the Gini index is a variance estimate of the distribution of class values in a node. In order to get the lowest Gini Index, all possible values for a split are being tested. The so called recursive binary splitting procedure described above needs to know when to stop splitting the training data and creating nodes. There are several methods to define a stop of splitting, but in the presented work the only rule was to use each feature in the optimised feature set only once. This is due to the fact that overfitting on the training dataset should be avoided.

## 3. Results

### 3.1. Typical Compensatory Movements

**Figure 5:**
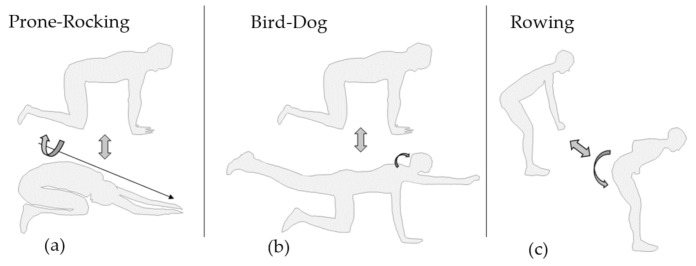
TCM: Exercises performed with a typical compensatory movement (**a**) Prone-Rocking, (**b**) Bird-Dog and (**c**) Bent-over Rowing.

Figure 5 shows the most critical typical mistakes occurring during performance of the three exercises as a result of the analysis that took place in collaboration with physiotherapists and orthopaedic surgeons. Figure 5a; during Prone-Rocking, patients tend to twist around the longitudinal axis of the spine in order to be able to touch the ground with one elbow. Figure 5b; When performing Bird-Dog, patients very often tilt their head upwards which results in a rotation around the transversal axis of the neck. Finally, Figure 5c illustrates the rounding of the lower back as the most common mistake when performing Bent-over Rowing, which can as well be described as a rotation around the transversal axis in the hip joint.

### 3.2. Feature Selection

The final feature combinations arising from the feature selection process for each exercise are shown in Table 3, Table 4, Table 5 and Table 6. Table 3 shows the best performing features for a single sensor scenario and Table 4, Table 5 and Table 6 when all sensors are used for the feature selection process. X, Y and Z each denote an axis and 1, 2 and 3 are the identifiers for the sensors (see Figure 1) on which the calculation was performed. For Bird-Dog, a combination of two features performed best in discriminating TCM from CPE. The first feature is the Root Mean Square of the Angles over time in Sensor 1 with respect to the gravity vector, φRMS1. The second feature is the Variance σ^2^ in the Z-axis of Sensor 3, called σ^2^Z3. For the exercise Prone-Rocking the variance in the Y-axis of Sensor 2 produced the best results and is called σ^2^Y2. In Rowing the best performing feature set consists of two features. First, the maximum between the angles of Sensors 1 and 2 with respect to the sensors’ gravity vectors, max(∆φ_1,2_). The second feature is the root mean square of the accelerations in the Z-axis of Sensor 3, called aRMSZ3.

Since the ultimate goal of this work is the support of patients in autonomously performing rehabilitation or prevention exercises, it might be useful to reduce the number of necessary sensors and additionally to focus on the simplest way to mount them. To analyse which sensor suits these needs best, Table 4 shows the accuracies and corresponding features when only one sensor is used.

Following the results in Table 3, Sensors two and three provide the best data for distinguishing between CPE and TCM in the three studied exercises. Furthermore, it seems to be advisable to categorise exercises by the best sensor position or to determine the best sensor position for each exercise separately.

### 3.3. Decision Trees

**Figure 6:**
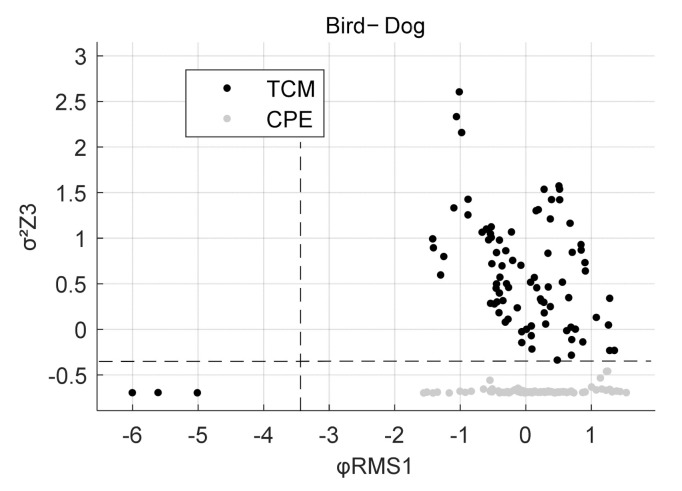
Scatter Plot for Bird-Dog with observations of TCM and CPE. Dashed lines at σ^2^Z3 = −0.39 and φRMS1 = −3.28.

Figure 6 displays the distribution of observations with σ^2^Z3 and φRMS1 for bird dog in a scatter plot. It can be seen that the split values are at −0.39 for σ^2^Z3 and −3.28 for φRMS1. The sensitivity as well as the specificity are at 98.3% (see Table 4).

**Figure 7:**
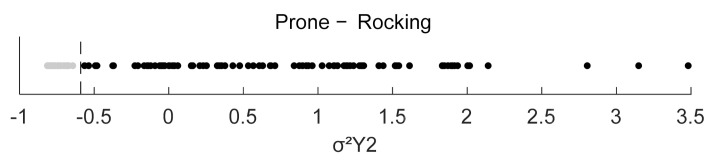
Scatter Plot for Prone-Rocking in one dimension. Dashed line at σ^2^Y2 = −0.62.

In Prone-Rocking for which the classifier only needs one feature for discriminating CPE from TCM, the split criterion is at −0.62 as the value for the variance in Sensor 2’s Y-axis. Figure 7 shows the corresponding one-dimensional scatter plot. In Table 5 the split value, the sensitivity and specificity for this classifier are documented.

**Figure 8:**
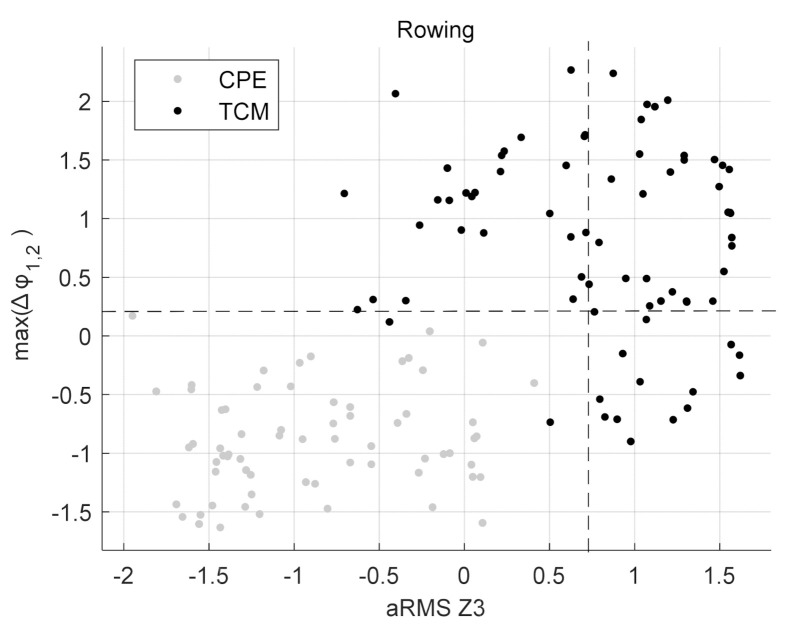
Scatter Plot for Rowing with observations of TCM and CPE. Dashed lines at max(∆φ_1,2_) = 0.27 and aRMSZ3 = 0.7.

The classifier for Rowing performs best when using the split values in Table 6: max(∆φ_1,2_) = 0.27, aRMSZ3 = 0.7. Figure 8 shows the corresponding scatter plot. With the specified split values, sensitivity and specificity both are at 82.8%.

## 4. Discussion

The aim of this work was to demonstrate the proof-of-concept of a method to detect typical compensatory movement in autonomously performed rehabilitation and prevention exercises aimed at LBP patients using three IMUs and thus facilitate guidance and control in community settings. Based on an analysis of 27 exercises that are typically prescribed to patients with LBP the three exercises Bird-Dog, Prone-Rocking and Rowing were used for the presented study. The analysis took place in collaboration with physiotherapists and orthopaedic surgeons to determine criteria for a clinically prescribed way of performing the exercises (CPE) and criteria for performing the exercises involving the most typical compensatory movements (TCM). In this study, the correlation between compensatory movements in rehabilitation or prevention exercises and accelerometer readings with a specified mounting procedure for the sensors was investigated. Identifying a set of features from inertial sensor data the method can distinguish CPE from TCM in the selected exercises. The study was also able to show that only one sensor is necessary to detect TCM with accuracies of 97.7%, 98.9% and 80.5% for Bird-Dog, Prone-Rocking and Rowing respectively.

Analysing the exercises in terms of movement axes was of great help to find an appropriate set of parameters. Bird-Dog for example, invokes a rotation around the longitudinal axis of the spine when being performed without compensatory movements. This movement may very well be impaired if a subject with limitations in hip mobility performs this exercise [7]. When the head is lifted an additional transversal rotation in the neck or extension of the cervical spine results in a hollow back which means an extension of the lumbar spine and an extended hip flexion. These movements can be seen in φRMS1. The transversal movement of the head is represented in σ^2^Z3. Furthermore, a rotation around the transversal axis of the shoulders is present in both CPE and TCM, but is not accounted for with the current sensor placement. In Prone-Rocking, the studied compensatory movement induces a rotation around the longitudinal axis along the spine which creates a rotational moment on the lumbar spine. Thus, it is intuitive that a feature in the second sensor—on the upper back—would be able to detect the deviation from the CPE best. However, the best performing feature here is based on the linear sideward acceleration σ^2^Y2 and not φRMS2 which derives from the tilt angle of the sensor. The studied TCM in Rowing (a round back through posterior pelvic tilt) can be observed by the relation of tilt angles of upper and lower back for which max(∆φ_1,2_) accounts best. The fact that aRMSZ3 contributes to the decision making might indicate that subjects tend to move their heads more up and down when performing a TCM compared to performing the CPE.

The exercise Rowing shows the limitations of the chosen sensor arrangement as TCM and CPE can only be distinguished with a maximum prediction accuracy of 82.8% and 76.4% when using only one sensor. The sensors used need to be moved at non-constant velocities to record accelerations. When assessing a subject while performing the exercise Rowing, the subject focused on only moving the arms. As such, this exercise was very likely to appear as static to the sensor system, which was on the not-moving back. An accuracy of 82.8% might still be considered a success but in the case of autonomous training scenarios, a user would not be effectively monitored. Additionally, the user would have to wear all of the three sensors in order to attain the maximal possible prediction accuracy.

In contrast to Morris et al. [10] the manually generated analogue signals are used to segment the recorded datasets into repetitions and no automated algorithms are implemented. This is beyond the scope of this work. Accordingly, prediction accuracies need to be treated on an exercise-specific basis.

As mentioned by Ranganathan et al. [23], movements exist on a continuum which provides researchers with the challenge of finding boundaries to distinguish compensatory from non-compensatory movement. This problem is further complicated when the movements are not known beforehand, which is likely happening in a community setting. As well as Ranganathan et al. this paper focuses on detecting compensatory movement in tasks that involve different combinations of movements using three wearable sensors and a maximum of two features. In addition to the acceleration sensor in this work, Ranganathan et al. used gyroscopes to record the angular velocities and reached values for precision and recall of 88.6% and 91.2% respectively. [23] The presented method reaches higher values which is mainly explainable through the different movements analysed and features used. Comparing both methods, the prediction accuracy is highly dependent of the representation of the underlying movement through the chosen feature parameters. However, it should be mentioned that the presented method only needs one acceleration sensor for proper detection of compensatory movements with accuracies of up to 98.3%.

The low number of subjects/observations and the fact that mostly exercise-savvy subjects were recruited might have resulted in insufficient amounts of data close to the class boundaries. On the other hand, the developed method has already shown potential to work properly for the case of preventing low back pain in healthy subjects who are under the risk of suffering from LBP. Thus, future work should consider recruiting healthy subjects who are not exercise-savvy to undermine the validation of the method that has been developed in this work. It should also be mentioned, that despite of the accurate instructions for performing each exercise the quality of exercise execution varied enough to result in realistic classification models for each of the three exercises.

Future work should consider patients rehabilitating from low back pain to analyse the accuracy of the method for detecting TCMs in this population.

It should be noted also that the training set in this study only trained for one TCM per exercise, similar to Eizentals et al. who used textile stretch sensors to cover all regions of the upper body in which strain was caused by the compensatory movements. This leads to the necessity that future work should also consider creating an extendable database of exercises and their corresponding typical compensatory movements that can be monitored. Although Eizentals et al. [24] analysed more exercises, they only recruited one subject.

Finally, a comparison of different classification algorithms could be carried out, to study the capabilities of classifying a wider range of different movements and thus combine the results of different studies. As the decision trees in the presented work have shown to be effective and easily explainable the presented method is interesting for clinical applications in the future.

## 5. Conclusions

This study was able to show that the monitoring of rehabilitation and prevention exercises is possible with only one sensor for two of the three test exercises. The accuracy in classifying CPE and TCM for the selected exercises of 97.7%, 98.3% and 76.4% for Bird-Dog, Prone-Rocking and Rowing respectively, using only sensor three (head) and a maximum of two features, showed that it was possible to detect the specified TCMs. This opens up the possibility of providing guidance and control for LBP related rehabilitation and prevention in community settings and prevents patients from movements that lead to a deterioration of their condition.

By being able to count the number of TCMs and determine the category of TCM (overextension of the neck, etc.), the opportunity arises to give detailed feedback to the patients. Additionally, this information enables therapists to advance patient-centred exercise plans, adjust them according to the patients’ development over time and determine physical stress due to the TCMs committed during exercise performance.

The presented method thus demonstrates an alternative to detect compensatory movement without the need of producing patient-specific shirts and with a single sensor that is easy to attach.

## Figures and Tables

**Table 1:** Overview of the process describing the subtasks of Generating Datasets, Data processing and Classification Model.

➀ Preparation	➁ Generating Datasets
Conduct experts on typical compensatory movements Define clinically prescribed exercise performance (CPE) Define typical compensatory movements (TCM)	Performance of clinically prescribed exercises (CPE) Performance of exercises involving typical compensatory movements (TCM) Detecting movement using accelerometers
**➂ Data Processing**	**➃ Classification Model**
Calculate parameter features Conduct feature selection algorithm Choose best performing sensor and corresponding feature CPE <−> TCM	Generate classification model with best performing features, sensors and explainability Test of the model

**Table 2:** Inclusion and exclusion criteria employed the selection of test subjects.

Inclusion Criteria	Exclusion Criteria
Subjects with affinity to movement between the ages of 18 and 35 years The subject is able to understand and perform the given exercises Functionally and anatomically fully preserved lower and upper extremities BMI ≤ 35 for better palpation	Pregnancy or lactation Epilepsy Diabetes Respiratory diseases Cardiovascular problems Low back pain Back related condition including trauma and surgery within last 5 years Current use of medication that affects coordination Existence of an allergic diathesis Physiotherapy within the last 3 months Hospital stay within the last three months

**Table 3:** Single sensor features and corresponding accuracies for binary decision trees.

	Bird-Dog		Prone-Rocking		Rowing		
	Parameter	Axis	Parameter	Axis	Parameter	Axis	
Sensor 1	max σ^2^	X Z	σ^2^	Y	aRMS φRMS	X	
Accuracy	67.0%		92.5%		69.0%		Ø 76.2%
	Parameter	Axis	Parameter	Axis	Parameter	Axis	
Sensor 2	φRMS σ^2^	X	σ^2^	Y	aRMS max	Z X	
Accuracy	94.3%		98.9%		80.5%		Ø 91.2%
	Parameter	Axis	Parameter	Axis	Parameter	Axis	
Sensor 3	σ^2^	Z	σ^2^	Y	aRMS σ^2^	Z X	
Accuracy	97.7%		98.3%		76.4%		Ø 90.8%

**Table 4:** Split Values, Accuracy, Sensitivity and Specificity for Bird-Dog Decision Tree calculated according to [43].

Feature				Split Value
Parameter	Sensor		Axis	
σ^2^	3		Z	−0.39
φRMS	1			−3.28
Accuracy		Sensitivity		Specificity
98.3%		98.3%		98.3%

**Table 5:** Split Values, Accuracy, Sensitivity and Specificity for Prone-Rocking Decision Tree calculated according to [43].

Feature				Split Value
Parameter	Sensor		Axis	
σ^2^	2		Y	−0.62
Accuracy		Sensitivity		Specificity
98.9%		100%		98.8%

**Table 6:** Split Values, Accuracy, Sensitivity and Specificity for Rowing Decision Tree calculated according to [43].

Feature				Split Value
Parameter	Sensor		Axis	
$\max\left(∆\varphi \right)$	1, 2			0.27
aRMS	3		Z	0.70
Accuracy		Sensitivity		Specificity
82.8%		82.8%		82.8%

## Data Availability

The datasets generated for this study are available on request to the corresponding author.
